# Aging Is Not Associated with Proteasome Impairment in UPS Reporter Mice

**DOI:** 10.1371/journal.pone.0005888

**Published:** 2009-06-11

**Authors:** Casey Cook, Jennifer Gass, Judith Dunmore, Jimei Tong, Julie Taylor, Jason Eriksen, Eileen McGowan, Jada Lewis, Jennifer Johnston, Leonard Petrucelli

**Affiliations:** 1 Mayo Clinic, Jacksonville, Florida, United States of America; 2 Elan Pharmaceuticals, South San Francisco, California, United States of America; National Institutes of Health, United States of America

## Abstract

**Background:**

Covalent linkage of ubiquitin regulates the function and, ultimately, the degradation of many proteins by the ubiquitin-proteasome system (UPS). Given its essential role in protein regulation, even slight perturbations in UPS activity can substantially impair cellular function.

**Methodology/Principal Findings:**

We have generated and characterized a novel transgenic mouse model which expresses a previously described reporter for UPS function. This UPS reporter contains a degron sequence attached to the C-terminus of green fluorescent protein, and is predominantly expressed in neurons throughout the brain of our transgenic model. We then demonstrated that this reporter system is sensitive to UPS inhibition *in vivo*.

**Conclusions/Significance:**

Given the obstacles associated with evaluating proteasomal function in the brain, our mouse model uniquely provides the capability to monitor UPS function in real time in individual neurons of a complex organism. Our novel mouse model now provides a useful resource with which to evaluate the impact of aging, as well as various genetic and/or pharmacological modifiers of neurodegenerative disease(s).

## Introduction

The ubiquitin-proteasome system (UPS) regulates the degradation of numerous regulatory proteins that control signal transduction, cell cycle progression and differentiation, as well as apoptotic pathways [1]. Ubiquitin ligases covalently link ubiquitin polypeptide chains to proteins, marking those proteins as substrates for the proteasome and allowing for targeted and selective degradation. In addition to degrading regulatory proteins, the UPS also degrades misfolded and damaged proteins, thus collectively implicating the UPS in a wide range of conditions, including neurodegenerative diseases, cancer, inflammation, and autoimmunity [2], [3].

The proteasome is a large, multisubunit complex containing a common proteolytic core, the 20S proteasome, which is composed of 28 subunits arranged in four, heptameric rings. The two outer rings are each composed of seven alpha-type subunits (α1–α7), while the two inner rings each contain seven beta-type subunits (β1–β7). The proteolytic activity is enclosed within the inner rings, with only the β1, β2, and β5 subunits possessing caspase-like, trypsin-like, and chymotrypsin-like cleavage specificity, respectively [4], [5]. The activity of the 20S proteasome is modulated by a variety of regulators, including the 19S/PA700 complex, PA200, as well as PA28 α/β and PA28γ [6]–[8]. The most common regulator, the 19S/PA700 complex, contains six AAA-family ATPases and is capable of binding both ends of the 20S proteasome in an ATP-dependent manner, forming the 26S proteasome, which is involved in the degradation of ubiquitinated proteins [9]–[11]. Given that only the 19S/PA700 complex possesses ATPase activity and binds to polyubiquitin chains, alternative regulators of the 20S proteasome are believed to modulate ubiquitin-independent functions of the proteasome.

A diverse group of neurological disorders that are characterized by an accumulation of ubiquitinated proteins (reviewed in [12]), suggesting that UPS dysfunction is likely to play a prominent role in the pathogenesis of neurodegenerative diseases. UPS impairment has been reported in aging [13], brain ischemia [14], [15], Huntington's disease (HD) [16]–[19], Cruetzfeldt-Jakob disease (CJD), Alzheimer's disease (AD) [20]–[23], Amyotrophic Lateral Sclerosis (ALS) [24], and Parkinson's disease (PD) [25]–[30]. Utilizing an innovative approach, Bedford and colleagues established a compelling link between dysfunction of the 26S proteasome and the development of α-synuclein neuropathology [31]. By genetically ablating a critical 19S/PA700 subunit (Rpt2/PSMC1) in the forebrain, and thus preventing formation of the 26S proteasome, Bedford and colleagues reveal that loss of 26S proteasome activity leads to synuclein and ubiquitin-positive inclusions in neurons of the forebrain, in addition to a learning deficit and progressive degeneration of forebrain regions. Intriguingly, as knockdown of Rpt2/PSMC1 expression leads to a specific impairment of 26S proteasome activity, while activity of the 20S proteasome is unaffected, neurodegeneration can be conclusively attributed to dysfunction of the 26S proteasome [31].

We have now generated a mouse model which will facilitate the identification of suitable targets in neurodegenerative diseases in which UPS impairment has been implicated. Development of therapies against these targets relies on our ability to pinpoint the exact stage of disease in which proteasome impairment contributes to the pathogenesis. To this end, we have engineered a transgenic mouse expressing a reporter (GFPμ) sensitive to perturbations in UPS function as demonstrated by Bence and colleagues [32]. In contrast to other transgenic mouse models expressing UPS reporters throughout the body [32], [33], GFPμ expression in our model is controlled by the mouse prion promoter (MoPrP). Use of the MoPrP for this UPS reporter system largely targets transgenic expression to neurons, providing an ideal model to evaluate the role of proteasome function in neuronal cell biology. In this report, we describe the development and characterization of this model, and determine that aging alone does not alter neuronal GFPμ expression in our mouse model.

## Results

### GFPμ protein and RNA is expressed throughout the brain in 2-month mice

The GFP-CL1 reporter proteasome substrate (GFPμ) was utilized to generate our transgenic model, termed Degron mice [34]. Briefly, GFPμ consists of a short degron sequence fused to the COOH end of the green fluorescent protein sequence, causing rapid ubiquitination and proteasomal degradation. High expression levels of this reporter, previously described by Bence and associates [32], are well tolerated in mammalian cells, and the system is very sensitive to the effects of proteasome inhibition.

**Figure 1:**
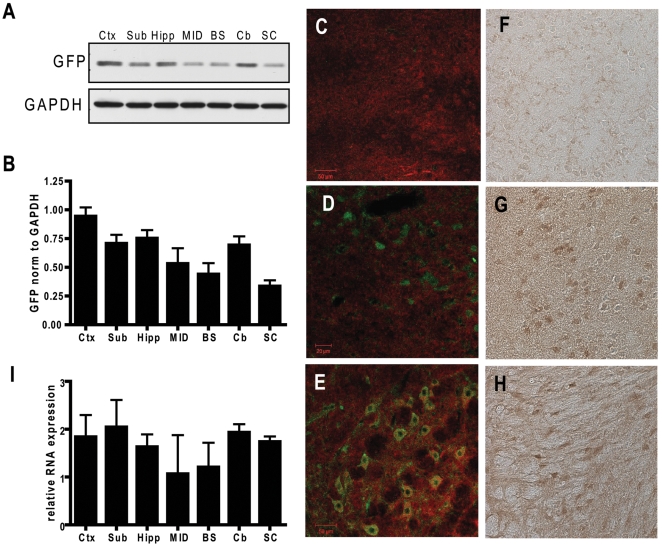
GFPμ protein and RNA is expressed throughout the brain. A) Representative WB showing regional GFPμ expression in a 2-month old GFPμ+/−mouse. B) Quantification of WB data performed by calculating GFP O.D. on brain regions from 4 mice heterozygous for GFPμ transgene, and normalizing values to GAPDH. A calibrator sample was included on each gel to compare protein expression across gels. C–E) Confocal microscopy of GFP immunoreactivity in nontransgenic cortex (C), and the cortex (D) and brainstem (E) from a 2-month old GFPμ+/−mouse (green = GFP, red = MAP2). F–H) Lack of GFP immunoreactivity in nontransgenic cortex (F), compared to specific GFP labeling in cortex (G) and brainstem (H) of a 2-month old GFPμ+/−mouse. I) Bar graph depicting quantification of regional mRNA expression from 4 heterozygous 2-month old GFPμ mice. (Ctx = cortex, Sub = subcortex, Hipp = hippocampus, MID = midbrain, BS = brainstem, Cb = cerebellum, SC = spinal cord; error bars = SEM).

Using western-blotting and qRT-PCR to evaluate regional GFPμ protein and mRNA transgene expression levels respectively in the Degron model, GFP is most highly expressed in cortical, subcortical, hippocampal, and cerebellar regions (see Figure 1A–B, I). Although the mRNA levels of GFP are relatively high in the spinal cord, GFP protein expression in the spinal cord is very low in comparison to other regions of the brain. Although native GFP fluorescence is preserved in free-floating tissue sections, it is difficult to detect GFP without antibody-mediated amplification of the GFP signal given the low level of GFPμ expression under conditions when proteasomal function is not impaired. Using immunohistochemical techniques, we were able to detect GFP in the GFPμ transgenic mice (Figure 1D–E, G–H), while specificity of the immunolabeling was demonstrated by the lack of immunoreactivity in non-transgenic mice (Figure 1C, F).

### Primary neuronal cultures from GFPμ mice are sensitive to proteasomal inhibition

**Figure 2:**
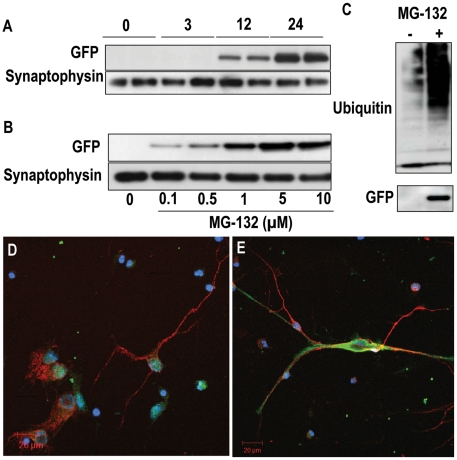
Primary neuronal cultures from GFPμ mice are sensitive to proteasomal inhibition. A) Induction of GFPμ following exposure to 5 µM MG132 is time dependent, with maximal induction observed at 24 hours. Synaptophysin was used as an endogenous loading control. B) GFPμ reporter is dose-dependently upregulated by 24 hours of MG132 treatment. C) Accumulation of GFPμ reporter is coincident with increased ubiquitination in the presence of MG132. D, E) Confocal microscopy of D = untreated and E = 5 µM MG132 for 24 hrs (red = MAP2, blue = DAPI, green = GFP).

To verify the sensitivity of the GFPμ reporter to proteasomal inhibition, as well as to monitor the kinetics of GFPμ induction, primary hippocampal neurons generated from GFPμ mice were treated with 5 µM MG132, a proteasomal inhibitor, and harvested at various time points after treatment (Figure 2A). As shown in Figure 2A, increased GFPμ protein levels are first observed at 12 hours, and maximal induction is observed following 24 hours of exposure to 5 µM MG132. In addition, primary hippocampal neurons from GFPμ mice exhibit a dose-dependent induction of GFPμ when exposed to various concentrations of MG132 (0.1 µM–10 µM) for 24 hours (Figure 2B). Upregulation of GFPμ is coincident with increased ubiquitination following proteasomal inhibition (Figure 2C), confirming the physiological relevance of GFPμ. Upregulation of GFPμ in response to proteasomal inhibition is also observed in primary hippocampal neurons using confocal microscopy (Figure 2D = untreated, 2E = 5 µM MG132 for 24 hours). We observed no difference in GFPμ mRNA levels between MG132-treated and untreated cells, confirming that the increase in GFPμ protein expression is due to a decrease in proteasome-dependent degradation and not influenced by translational changes (data not shown).

### GFPμ mice are sensitive to proteasomal inhibition *in vivo*

**Figure 3:**
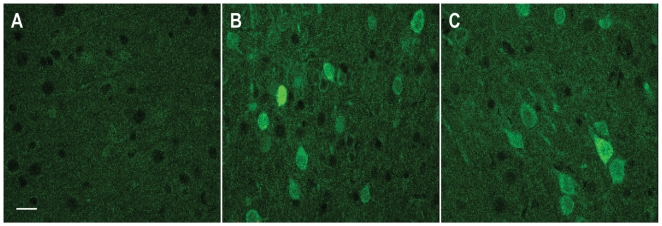
GFPμ mice are sensitive to proteasomal inhibition *in vivo*. A) Without antibody-mediated amplification, little GFP immunofluorescence is observed in cortical neurons near injection site of vehicle-injected control. B, C) In contrast, GFP immunofluorescence is easily detectable and significantly upregulated in cortical neurons near injection site of MG132-injected mouse. Scale bar, 20 µm.

To validate the GFPμ reporter *in vivo*, we performed stereotaxic injections of the proteasomal inhibitor MG132 into the cortices of GFPμ mice. Confirming our observations from primary neuronal cultures, injection of MG132 led to a prominent increase in cortical GFPμ expression (Figure 3). In addition, given the extent of GFPμ upregulation in the MG132-injected mice, it was possible to detect GFPμ without antibody-mediated amplification of the GFP fluorescent signal (Figure 3B–C). We detected only minimal native GFP fluorescence in vehicle-injected (Figure 3A) and uninjected controls (data not shown).

### Effect of aging on GFPμ protein and RNA expression

**Figure 4:**
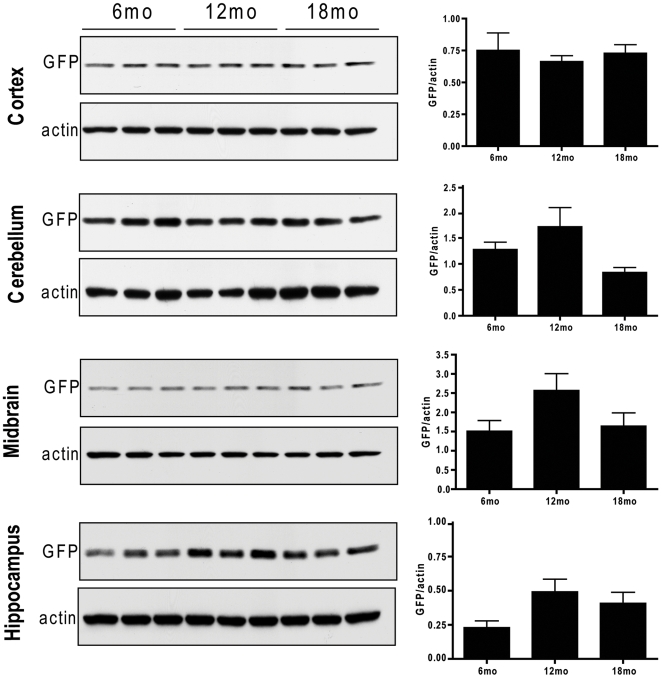
No effect of aging on GFPμ protein expression. Representative WB showing GFPμ expression in cortex (Ctx), cerebellum (Cb), midbrain (MID), and hippocampus (Hipp) from 6 to 18 months of age in heterozygous GFPμ mice. Quantification of GFP O.D. was normalized to actin to control for protein loading (each bar represents average GFP expression for *n* = 6 mice [3 males, 3 females] at each time point, with error bars depicting SEM).

**Figure 5:**
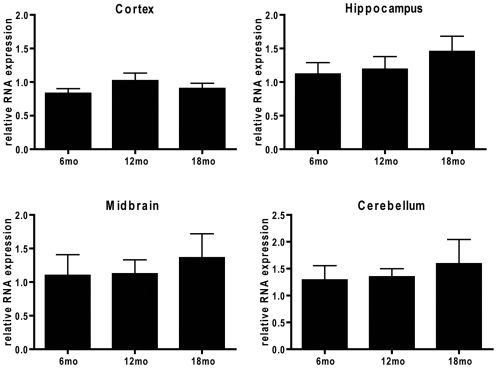
No effect of aging on GFPμ RNA expression. Regional GFPμ mRNA expression was evaluated using GAPDH as an endogenous loading control for each sample, and the lowest expressing sample for each region used as the calibrator sample for that respective brain region. Samples were loaded in quadruplicates, and an *n* = 6 mice used to determine RNA expression at each time point. Quantification of relative mRNA expression revealed no effect of aging in any of the brain regions evaluated (cortex, F = 0.963, *p* = 0.409; hippocampus, F = 0.831, *p* = 0.456; cerebellum, F = 0.265, *p* = 0.771; midbrain, F = 0.22, *p* = 0.806).

Given that aging has been reported to lead to UPS impairment [13], we evaluated GFP protein (Figure 4; Figure S1) and mRNA expression (Figure 5) in aged cohorts of Degron mice to determine if aging alone would lead to an increase in GFP expression. Surprisingly, as demonstrated in Figure 4, there was no change in GFP protein expression between the ages of 6 to 18 months in the cortex (F = 0.243, *p* = 0.788), hippocampus (F = 3.092, *p* = 0.075), midbrain (F = 2.598, *p* = 0.108), or cerebellum (F = 3.377, *p* = 0.062), which is consistent with results from immunohistochemical studies on aging GFPμ mice (Figure S1). In addition, there was no observed difference in GFP accumulation with age between male and female GFPμ mice. As shown in Figure 5, there was also no effect of aging on regional GFPμ mRNA expression (cortex, F = 0.963, *p* = 0.409; hippocampus, F = 0.831, *p* = 0.456; cerebellum, F = 0.265, *p* = 0.771; midbrain, F = 0.22, *p* = 0.806), indicating that aging alone does not significantly impair proteasomal function in our mouse model.

## Discussion

In this report, we describe the development, characterization, and validation of our novel UPS reporter transgenic mouse model. In contrast to earlier reports [13], we show that aging is not associated with proteasome impairment in our mouse model, though it is possible we may observe UPS dysfunction in mice aged beyond 18 months [13]. However, our finding is consistent with a recent study by the Dantuma lab using a transgenic mouse expressing a different UPS reporter substrate [24]. Specifically, the Dantuma group employed a model expressing a mutant form of ubiquitin (G96V) fused to the N-terminus of GFP, which cannot be cleaved and is instead polyubiquitinated on both the lysine 29 and 48 residues and targeted for proteasome-mediated degradation [24], [35]. Given that two different UPS reporter substrates do not exhibit a significant accumulation with age, it is possible that previous work evaluating proteasome activity and subunit expression in mouse brain homogenates does not accurately reflect UPS function *in vivo* [13], making models such as ours valuable in elucidating the *in vivo* roles of the UPS. Additionally, the recent demonstration that specific inhibition of the 26S proteasome leads to a neurodegenerative phenotype [31], as well as the finding that neurons and glia display different basal levels of UPS activity [36], *in vivo* models expressing UPS reporters are becoming increasingly more valuable. These recent studies also highlight the major shortcomings of earlier reports assessing proteasome function in brain homogenates utilizing enzymatic assays, which do not discriminate between 26S versus 20S proteasomes, and also cannot differentiate between various cell types.

The utility of UPS reporter mice has also increased in parallel with the number of neurodegenerative diseases being linked to UPS dysfunction, making it somewhat of a common theme associated with neurodegeneration. For example, soluble Aβ oligomer formation and impaired proteasomal function are observed concomitantly in the triple transgenic mouse model of AD; however, proteasome activity is restored when soluble Aβ oligomers are converted into insoluble aggregates [23]. This suggests that soluble oligomeric Aβ species, and not the monomeric or fibrillar form of Aβ, inhibits proteasomal activity [23]. UPS impairment has also been observed in cell culture and animal models overexpressing mutant huntingtin protein [16]–[19], , and consistent with data proposing a protective effect of aggregation due to sequestration of toxic species, treatment with a compound that increases inclusion formation prevents huntingtin-mediated proteasome inhibition [19]. However, the lack of GFP accumulation in an HD mouse model (R6/2) crossed to our GFPμ mice may be indicative of a complex relationship between overexpression of mutant polyglutamine proteins and UPS function [40]. Importantly for prion disorders such as CJD, the abnormal prion conformer (PrP_sc_) inhibits the 26S proteasome *in vitro*, while either preincubation with an oligomer antibody or heat denaturation of PrP_sc_ alleviated this inhibitory effect [22]. These findings indicate that a specific conformation of an oligomeric PrP_sc_ intermediate mediates the proteasomal inhibitory effect [22]. Proteasome activity was also significantly decreased in both cells exposed to prion-infected mouse brain homogenates and in brain regions exhibiting significant prion neuropathology in mice infected with PrP_sc_. This finding establishes a solid link between UPS impairment and neurodegeneration associated with prion infection [22].

In regard to the link between UPS function and PD, multiple publications have consistently reported greater proteasomal impairment in the presence of aggregated α-synuclein in comparison to its monomeric counterpart [41]–[43]. Although both monomeric and aggregated α-synuclein have been shown to bind the S6′/TBP1 (Tat binding protein 1) subunit of the 19S/PA700 proteasome complex [42], [44], only aggregated α-synuclein inhibits ubiquitin-dependent and independent 26S proteasomal activity [42]. Zhang and colleagues have demonstrated that α-synuclein protofibrils inhibit the ubiquitin-independent degradation of unstructured proteins by the 26S proteasome, though monomers and dimers have no effect on the proteolysis of these substrates [43]. In contrast, ubiquitin-dependent 26S proteasome activity is slightly inhibited by monomeric and dimeric α-synuclein, while protofibrillar α-synuclein potently inhibits the degradation of polyubiquitinated proteins. Given that α-synuclein protofibrils bind the 19S/PA700 regulatory complex of the 26S proteasome, as well as p21 (an unstructured proteasomal substrate) and K48-linked polyubiquitin chains, it is proposed that α-synuclein protofibrils inhibit 26S proteasome activity by interfering with substrate translocation into the proteasome core, achieved through direct interactions with the proteasome, as well as through the sequestration of proteasomal substrates [43], [45].

The GFPμ UPS reporter mice described in this report provide a novel resource with which to monitor the activity of the 26S proteasome *in vivo*. A number of mouse models are available for the neurodegenerative diseases in which UPS dysfunction has been implicated. Crossing our Degron model with these neurodegenerative disease models, such as those for AD, will allow a unique opportunity to directly follow the UPS impairment in relation to neuropathological, biochemical, and behavioral features of the model system. Such studies should help investigators understand if and when the UPS plays a key role in disease pathogenesis. Ultimately, such findings with our transgenic mouse model could be utilized to identify key therapeutic timepoints in the disease process, and to develop and screen therapeutic agents for the potential to modulate UPS function at these timepoints.

## Materials and Methods

### Generation of GFPu construct/mice

To generate transgenic mice carrying the GFPμ reporter (described in [34]), the GFP-CL1 sequence was cloned into the *Eco*R1 site of the mouse prion promoter construct [46]. The construct cDNA was linearized with *Not*I, gel purified, and injected into the pronuclei of fertilized eggs harvested from C57BL6/DBA2/SW mice. Founder mice were bred to C57BL6 mice, and progeny were screened at 3 weeks of age for integration of the transgene using PCR and dot blotting analysis of genomic DNA obtained from tail biopsies. 10 founder lines were expanded for further characterization, and the founder line with the highest level of expression was then used for all subsequent experiments.

### Genotyping/GFP primers

To genotype mice, tail biopsies from 3 week old mice were digested overnight at 55°C in Direct PCR Tail lysis buffer (Qiagen) and proteinase K (Qiagen). Samples were then cooled to room temperature, and PCR performed using GFP specific primers (5′GTG ACT CGA GAG ATC CGC TAG CGC TAC C 3′) and (5′ CAC CTT GAT GCC GTT CTT CT 3′) detecting a 0.5 kB GFP band, against an internal 0.3 kB β-actin control (5′ CGG AAC CGC TCA TTG CC 3′) and (5′ ACC CAC ACT GTG CCC ATC TA 3′). Tail DNA combined with dH2O, 10× PCR buffer (Qiagen), Q solution, each of the four primers, dNTPs, and Taq (5 U/µl; Qiagen). DNA samples were then denatured for 4 minutes at 94°C using an Eppendorf mastercycler (epgradient S model), undergo 3 cycles of 94°C for 15 seconds, 65°C for 30 seconds, and 72°C for 45 seconds, 10 cycles of 94°C for 15 seconds, 65°C for 30 seconds, and 72°C for 30 seconds, 20 cycles of 94°C for 15 seconds, 60°C for 30 seconds, and 72°C for 45 seconds, before a final 72°C extension step for 10 minutes.

### Primary neuronal culture

For primary neuronal cultures, hippocampi from postnatal day 2 mouse pups were removed and stored at 4°C in HIBERNATE™ A media without calcium (BrainBits), supplemented with B27 (Invitrogen), 0.5 mM GMAX (GIBCO), and gentamicin (GIBCO). Excised hippocampi were digested in papain (2 mg/mL; Fisher Scientific), triturated with a Pasteur pipet (bore size 0.8–1 mM), centrifuged to collect cell pellet, and resuspended in Neurobasal A (Invitrogen), supplemented with B27, GMAX, gentamicin, and bFGF (Invitrogen). Following determination of cell number, neurons were plated on poly-D-lysine-coated coverslips within 24-well plates for immunocytochemical studies (seeded at a density of 2.5×10^4^ cells/coverslip), or seeded on poly-D-lysine coated 6-welll plates for immunoblotting at a seeding density of 2.5×10^5^ cells/well.

### Mouse sacrifice/harvest

For immunohistochemical studies, mice were terminally anesthetized with sodium pentobarbital (i.p. 45–80 mg/kg), and when unresponsive to toe pinch, a longitudinal incision was made over the sternum, the diaphragm and ribs were cut, and the sternum peeled back to expose the heart. A 25-gauge butterfly needle attached by tubing to a perfusion pump was placed in the left ventricle, and an incision made in the right atrium. The mice were then transcardially perfused with saline (2.25 mL/min for 5 minutes until all blood was flushed from the system), followed by fixative (4% paraformaldehyde; approximately 2.25 mL/min for 15 minutes). Brains and spinal cords were then removed and placed in 4% paraformaldehyde for 24 hours, and cyropreserved in 30% sucrose until sectioning.

For regional protein and mRNA analysis, mice were sacrificed by CO_2_ asphyxiation, and brains were rapidly removed and both halves dissected on ice into 6 regions (cortex [CTX], subcortex [SUB; includes striatum, thalamus, and hypothalamus], hippocampus [HIPP], midbrain [MID], brainstem [BS], cerebellum [CB]). Spinal cords were also excised, and all samples frozen on dry ice.

### Immunohistochemistry/immunofluorescence and confocal microscopy

Fixed brains were sectioned at 40 microns on a sliding microtome (Leica SM2400), and stored in 0.12 M PBS with 0.02% sodium azide. Free-floating sections were washed in 0.05 M PBS with 0.02% triton X (PBS-Tx), blocked for 1 hour in 10% normal goat serum (PBS-Tx), and incubated overnight in anti-MAP2 (1∶500, Sigma-Aldrich) or anti-GFP (1∶100, Chemicon) diluted in 1% normal goat serum (PBS-Tx). On the following day, sections were washed in PBS-Tx, incubated for 1 hour in goat anti-rabbit Alexa Fluor 568 (1∶1000, Molecular Probes) or goat anti-mouse Alexa Fluor 488 (1∶1000, Molecular Probes) diluted in 1% normal goat serum (PBS-Tx). Sections were then washed in PBS-Tx, incubated for 10 minutes in Hoescht 33258 (1∶10000, Invitrogen) diluted in PBS-Tx. Sections were again washed in PBS-Tx, and then mounted onto Superfrost plus microscope slides (Fisherbrand). After sections were dry, slides were coverslipped with Fluoromount G (Southern Biotech). The depicted GFP immunofluorescence from the MG132-stereotaxic injections into the cortex of GFPμ mice did not require antibody amplification.

### Immunoblotting procedures

Each of the 6 sub-dissected regions from the left hemisphere and the spinal cord were weighed and homogenized in 10× volume of homogenate buffer (50 mM Tris-HCl [pH 7.4], 300 mM NaCl, 5 mM EDTA, 1% Triton-X-100, 1% SDS, 1 mM PMSF, protease inhibitor cocktail, phosphatase inhibitors I and II [Fisher Scientific]). Following sonication, samples were centrifuged at 16,000 g for 15 minutes, and a BCA protein assay (Thermo Scientific) performed on the supernatant. 30 µg of protein from each sample was diluted in dH_2_O, 2× tris-glycine SDS sample buffer (Invitrogen), and 5% β-mercaptoethanol (Sigma-Aldrich), and heat-denatured for 5 minutes at 95°C. Samples were run on 4–20% tris-glycine gels (Invitrogen), and transferred to PVDF membrane (Millipore). Membranes were blocked in 5% milk in TBS/0.1% Triton-X-100, and incubated overnight in anti-GFP (1∶2000; Invitrogen), anti-actin (1∶10,000; Sigma), or anti-GAPDH (1∶10,000; BioSource) at 4°C. Membranes were incubated in HRP-conjugated secondary antibodies (1∶5000; Jackson Immuno) for 1 hour at room temperature, and detected by ECL (PerkinElmer).

### qRT-PCR

Total RNA was extracted from the 6 brain regions (right hemisphere) and spinal cord using the TRIzol/Total RNA Purification System (Invitrogen). Concentration of RNA was determined using Nanodrop, and 0.5 ug RNA was converted to cDNA using the SuperScript III First-Strand Synthesis System (Invitrogen). Real-time PCR was performed on an ABI7900 using SYBR green (Applied Biosystems) as the detector. Samples were run in quadruplicate, with GAPDH used as an endogenous calibrator for each sample. The SYBR green fluorescent signal was analyzed using SDS2.2.2 software, and relative quantities of GFPμ were determined.

### Stereotaxic surgical procedures

For stereotaxic injections, 1 month old GFPμ transgenic mice were anesthetized with isoflourane (3% for induction, 1.5% for maintenance) and deemed anesthetized when the corneal eyeblink and ear-twitch reflexes could no longer be elicited by touch. Mice were then placed in a Kopf stereotaxic instrument, and the scalp cleaned with iodine and isopropanol. A midsagittal longitudinal incision was made in the scalp to expose the skull, and two small burr holes drilled through the skull (from Bregma, anterior posterior −1.6, mediolateral ±1.5, dorsal-ventral −1.5). A 10 µL Hamilton syringe mounted in an UMP2 Microsyringe injector and Micro4 Pump (World Precision Instruments, Sarasota FL) on the Kopf apparatus was inserted into the right cortex, and 2 µL of vehicle (10% DMSO) or MG132 (5 mM in 10% DMSO) injected into the brain at a flow rate of 0.2 µL/min. The procedure was repeated to inject vehicle/MG132 into the left cortex. Following the second injection, Michel clips were used to close the scalp, and mice were injected with sterile saline for hydration, and placed on a hot pad under a heating lamp for 2 hours. Mice were also administered acetaminophen in gelatin ad libitum both pre- and post-operatively for pain management, and monitored for signs of distress. Mice were sacrificed by CO_2_ asphyxiation 24 hours post-injection. Aseptic techniques were used for all surgical procedures, and all mouse procedures were performed under an approved IACUC protocol and in accordance with guidelines established by the NIH.

## Supporting Information

Figure S1 No effect of aging on GFP immunolabeling in GFPμ mice. GFP immunoreactivity in cortex (Ctx), midbrain (MID), and hippocampus (Hipp) from 2 to 18 months of age in heterozygous GFPμ mice. Magnification, 10×; inset, 40×.
(15.31 MB TIF)
