# Supplementary figures and images for: Aging Is Not Associated with Proteasome Impairment in UPS Reporter Mice
Source: PLoS One. 2009 Jun 11;4(6):e5888. doi: 10.1371/journal.pone.0005888 (PMC2690827; doi:10.1371/journal.pone.0005888)

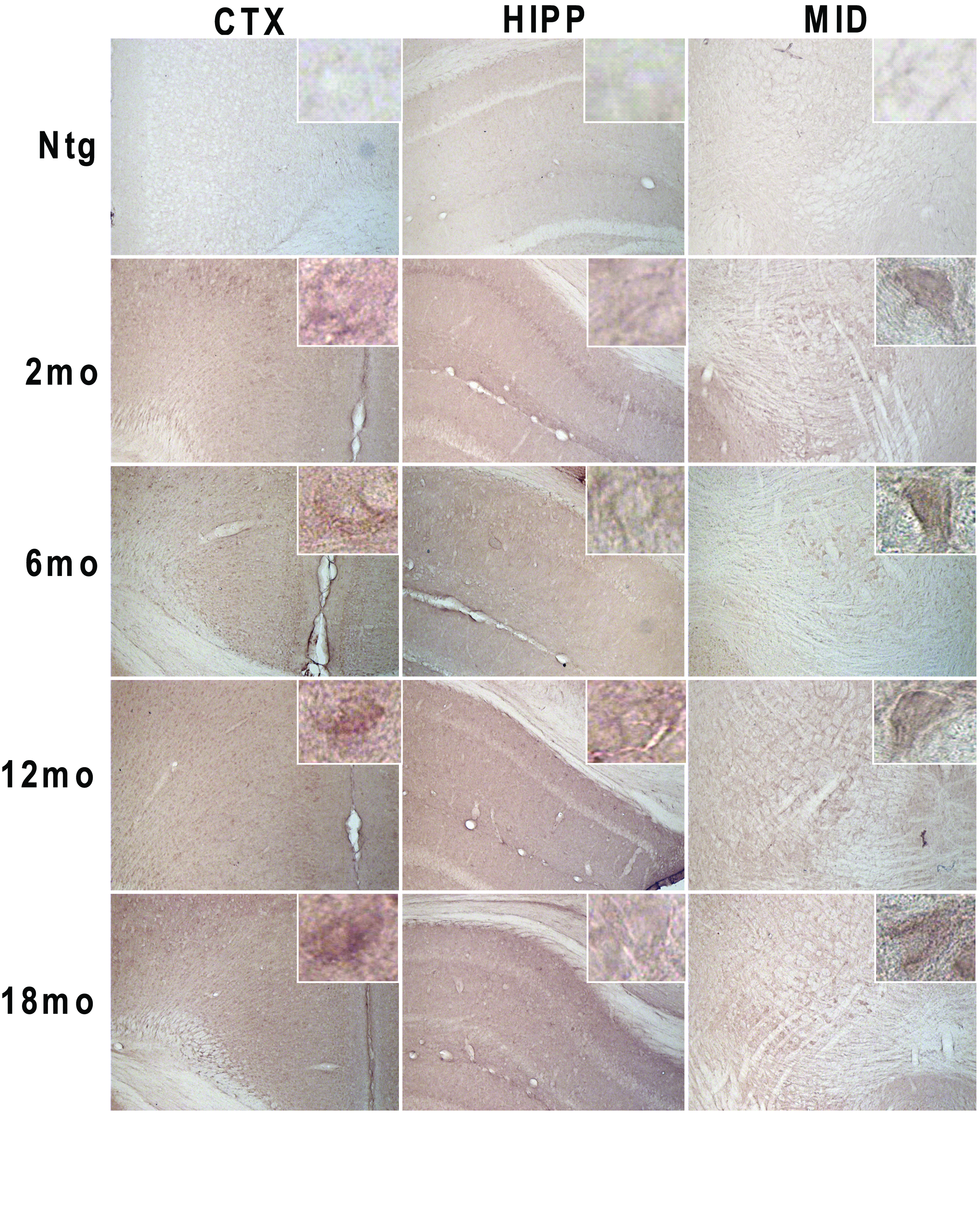

Supplement: Figure S1 — No effect of aging on GFP immunolabeling in GFPμ mice. GFP immunoreactivity in cortex (Ctx), midbrain (MID), and hippocampus (Hipp) from 2 to 18 months of age in heterozygous GFPμ mice. Magnification, 10×; inset, 40×. (15.31 MB TIF) [file pone.0005888.s001.tif]
